# GTSP1 expression in non-smoker and non-drinker patients with squamous cell carcinoma of the head and neck

**DOI:** 10.1371/journal.pone.0182600

**Published:** 2017-08-17

**Authors:** Pamela de Oliveira Soares, Patrícia Maluf Cury, Rossana Verónica Mendoza López, Cláudio Roberto Cernea, Erika Erina Fukuyama, David Livingstone Alves Figueiredo, Francisco Gorgonio da Nobrega, Otavio Alberto Curioni, Fabio Daumas Nunes, Raquel Ajub Moyses, Maria Lúcia Bueno Garcia, on behalf of HEAD AND NECK GENOME PROJECT–GENCAPO

**Affiliations:** 1 Department of Head and Neck Surgery, Hospital das Clinicas da Faculdade de Medicina da Universidade de São Paulo / LIM-28—São Paulo, SP, Brazil; 2 Department of Pathology and Legal Medicine–Faculdade de Medicina de São José do Rio Preto São José do Rio Preto, SP, Brazil; 3 Center for Research in Oncology, ICESP- Instituto do Câncer do Estado de São Paulo, SP, Brazil; 4 Department of Head and Neck Surgery, Instituto do Câncer Arnaldo Vieira de Carvalho, São Paulo, SP, Brazil; 5 Department of Pharmacy, Universidade Estadual do Centro-Oeste, Guarapuava, PR, Brazil; 6 Institute of Science and Technology, Universidade Federal de São Paulo, São José dos Campos, SP, Brazil; 7 Department of Otorhinolaryngology and Head and Neck Surgery, Hospital Heliópolis São Paulo, SP, Brazil; 8 Department of Oral Pathology Faculdade de Odontologia da Universidade de São Paulo, São Paulo, SP, Brazil; 9 Department of Internal Medicine, Hospital das Clinicas da Faculdade de Medicina da Universidade de São Paulo, São Paulo, SP, Brazil; National University Singapore Yong Loo Lin School of Medicine, SINGAPORE

## Abstract

**Introduction:**

The main risk factors for head and neck squamous cell carcinoma (HNSCC) are tobacco and alcohol consumption and human papillomavirus (HPV) infection. However, in a subset of patients, no risk factors can be identified. Glutathione S-transferase π (GTSP1) is a carcinogen-detoxifying enzyme that is activated by exposure to carcinogens, and it is associated with a reduction in response to toxic therapies. We studied the expression of GTSP1 in tumor and non-tumor tissue samples from patients with and without these risks to identify whether GTSP1 expression differs according to exposure to carcinogens.

**Materials and methods:**

Non-smoker/non-drinker (NSND) and smoker/drinker (SD) patients were matched according to age, gender, tumor site, TNM stage, grade and histological variants to establish 47 pairs of patients who have been previously tested for HPV. GTSP1 immunostaining was analyzed using a semi-quantitative method with scores ranging from 0 to 3 according to the area of immunostaining.

**Results:**

GTSP1 expression was detected in the tumors of both groups. GTSP1 expression was higher in the non-tumor margins of SD patients (*p = 0*.*004*). There was no association between GTSP1 expression and positivity for HPV. No differences in survival were observed according to GTSP1 staining in tumors and non-tumor margins.

**Conclusion:**

This study showed that GTSP1 was expressed in tumors of HNSCC patients regardless of smoking, drinking or HPV infection status. The difference in GTSP1 expression in non-tumor margins between the two groups may have been due to two possible reasons. First, elevated GTSP1 expression in SD patients might be the result of activation of GTSP1 in response to exposure to carcinogens. Second, alternatively, impairment in the detoxifying system of GTSP1, as observed by the reduced expression of GTSP1, might make patients susceptible to carcinogens other than tobacco and alcohol, which may be the underlying mechanism of carcinogenesis in the absence of risk factors.

## Introduction

Head and neck squamous cell carcinoma (HNSCC) is a major health problem worldwide. Tobacco and alcohol are the main risk factors of HNSCC in addition to human papillomavirus (HPV) infection [1]. However, in a small but increasing subset of patients, no risk factors can be identified, indicating a possible role of environmental and/or genetic factors in cancer development.

Recent studies [2–4] have demonstrated that genetic polymorphisms that impair the activity of detoxifying enzymes might contribute to carcinogenesis. One of the main systems of cellular detoxifying enzymes consists of glutathione S-transferases (GSTs), a superfamily of phase II enzymes that participate in the detoxification of carcinogens, including tobacco and alcohol [5–9].

Glutathione S-transferases π (GTSP1) is one of the GSTs that are usually expressed in HNSCC [10]. It has also been implicated in resistance to cytotoxic treatment modalities in cancer [11, 12], as it detoxifies chemotherapeutic compounds and products of oxidative stress generated by radiotherapy [13–15]. Low expression of GTSP1 may be associated with better treatment responses and better prognosis [16].

It is unknown whether an increase in expression of GTSP1 in HNSCC is a consequence of persistent exposure to tobacco and alcohol, which is frequently observed in patients with HNSCC, or whether GTSP1 is activated by other carcinogenic mechanisms in these tumors. This can impact the use of GTSP1 as a possible predictor of treatment response and prognostic marker in HNSCC patients who are not exposed to alcohol and tobacco. Prediction of disease response and prognosis should be differentially evaluated according to the smoking and drinking habits of patients [17]. However, no study on GTSP1 has been conducted specifically in non-smoker/non-drinker (NSND) patients. These data could also clarify whether the impairment of the detoxifying effect of GTSP1 could be one of the mechanisms underlying the incidence of HNSCC in NSND patients. Thus, the aim of this study was to compare the expression of GTSP1 in tumor and non-tumor tissue samples from patients with HNSCC according to their smoking and drinking habits. We also analyzed the prognostic value of GTSP1 expression in patients who were exposed to tobacco or alcohol, since both these habits are important predictors of survival and can act as confounders in the analysis of the prognostic value of GTSP1. Additionally, our group of smoker/drinker (SD) patients were matched to a selected subset of NSND patients and are thus not representative of all SD patients.

## Materials and methods

Patients older than 18 years with squamous cell carcinoma of the oral cavity (lips excluded), oropharynx, larynx and hypopharynx without previous treatment were prospectively enrolled from January 2001 to February 2009 in the head and neck surgery departments of five hospitals of the Brazilian Head and Neck Genome Project, Gencapo, a collaborative consortium of research groups from hospitals and universities in the state of São Paulo, Brazil.

### Ethics statement

All clinical investigations were conducted according to the principles in the Declaration of Helsinki. Written informed consent was obtained from each participant during study enrollment. The study was approved by the Ethics Committee of the Hospital das Clinicas, University of São Paulo under the protocol number 0511/07. All materials provided to the research team were de-identified.

Among the 1633 patients included, 80 were considered NSND [18]. Patients were considered to be smokers if they had smoked at least one cigarette, cigar, or pipe daily for at least one year during their lifetime and as drinkers if they had consumed alcoholic beverages at least once a month on a regular basis [19]. SD patients were selected as matched pairs of NSND patients according to gender, age (+/- 5 years) tumor site, clinical stage, tumor degree and histological variants. Tumor staging was performed according to the 5th (until 2002) and 6th editions (since 2003) of the American Joint Committee on Cancer (AJCC) TNM classification criteria [20, 21].

Formalin-fixed, paraffin-embedded (FFPE) samples were obtained from all NSND patients and corresponding SD pairs, which included both tumors and non-tumor margins, to evaluate GTSP1 expression via immunohistochemistry (IHC).

Previous data on HPV were also retrieved for the patients using PCR [22] and analyzed according to GTSP1 expression in both SD and NSND patient groups.

### Immunohistochemical assay

Consecutive 3 μm-thick sections were cut from each paraffin block and mounted οn glass slides pre-treated with 3-aminopropyl triethoxysilane/acetone solution. Following deparaffinization, the sections were re-hydrated, treated with citrate buffer at 96°C for 30 min and further treated with 3% H2O2 in methanol (v/v) for 30 min to block endogenous peroxidases. To block non-specific binding, the slides were treated with Background Sniper from Starr Trek Detection kit (Biocare, California, USA) for 10 min. The sections were then incubated for 16 hours at 4°C with the specific antibody Novocastra NCL-L-GTSP1-437 (Leica Biosytems, UK). The slides were incubated with biotinylated secondary antibody and then with streptavidin-biotin peroxidase following the manufacturer’s instructions (Kit Starr Trek Detection, Biocare, California, USA). The immunostain was visualized with 3,3’-diaminobenzidine tetrahydrochloride (DAB) containing 0.005% H2O2 and counterstained with hematoxylin. Negative controls were established by replacing the primary antibody with buffer. The slides were analyzed using light microscopy (400× magnification).

Negative controls were used in all steps. Tissue samples known to be positive for GTSP1 were used as positive controls.

Two of the authors (POS and PMC), who were blinded to the clinical data, quantified GTSP1 expression in the entire area of each sample in a semi-quantitative fashion. The expression was ranked as follows: 0 in case of absence of staining or staining in less than 10% of cells; 1 for staining in 10% to 30% of cells; 2 for staining in 31% to 60% of cells; and 3 for staining in more than 60% of cells [23]. The categories were further merged into low (0 and 1) and high (2 and 3) GTSP1 expression to facilitate statistical analyses [24].

### Statistical analysis

Fisher’s exact test was used to test the association between HPV and GTSP1 for all patients. The data on non-tumoral and tumoral GTSP1 expression and HPV positivity among pairs were analyzed using McNemar’s test. Overall survival was analyzed only for NSND patients. Survival was determined from the end of treatment until death or loss of follow-up and analyzed using Kaplan-Meier curves and the log-rank test.

A two-tailed p-value of 0.05 was considered significant. No adjustments for multiple comparisons were made since the exploratory nature of the study. Statistical analyses were performed on SPSS for Windows v. 18.

## Results

Among the 80 NSND patients, it was possible to find matched SD patient counterparts for 47. In the 47 matched pairs, there were 47 NSND and 37 SD patients. For this reason, some SD patients were matched to more than one NSND patient based on the principle of hot deck imputation [25]. Table 1 displays the clinical features of the paired samples. Most of the patients were female (62%) and presented with oral cavity tumors.

**Table 1:** Clinical features of the paired samples.

Feature	NSND	SD
	N = 47	N = 47
	N (%)	N (%)
**Age**		
Median (range)	65 (35–83)	68 (39–80)
**Site**		
Oral cavity	33 (70.2)	33 (70.2)
Oral cavity + oropharynx	2 (4.3)	2 (4.3)
Oropharynx	1 (2.1)	1 (2.1)
Larynx	10 (21.3)	10 (21.3)
Hypopharynx	1 (2.1)	1 (2.1)
**T stage**		
T_1_	10 (21.3)	16 (34.0)
T_2_	18 (32.3)	10 (21.3)
T_3_	6 (12.8)	4 (8.5)
T_4_	13 (27.7)	17 (36.2)
**N stage**		
**N**_**0**_	29 (61.7)	29 (61.7)
N+	18 (38.3)	18 (38.3)
**Differentiation**		
Well differentiated	28 (62.2)	22 (46.8)
Moderately differentiated	16 (35.6)	24 (51.1)
Poorly differentiated	1 (2.2)	1 (2.1)
**Tumor variants**		
Squamous cell carcinoma	41 (97.6)	42 (91.3)
Verrucous carcinoma	0	
Basaloid	1 (2.4)	4 (8.7)

NSND: Non-smoker/non-drinker; SD: smoker/drinker

It was possible to perform successful GTSP1 immunostaining for tumor samples from 30 pairs of patients and for non-tumor samples from 16 pairs of patients. No histological features of pre-malignant lesions were detected in any of the non-tumor margins.

**Fig 1:**
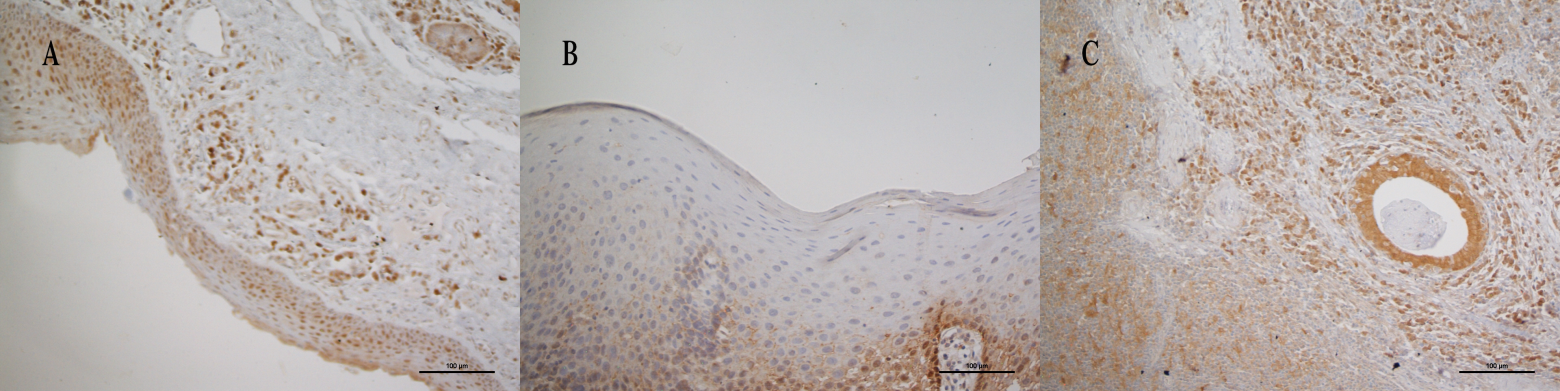
Expression of GTSP1 via immunohistochemistry. A: High expression of GTSP1 in non-tumor margin; B: low expression of GTSP1 in non-tumor margin; C: high expression of GTSP1 in tumor.

Fig 1 represents the examples of different GTSP1 expression patterns in tumor and non-tumor samples. Table 2 presents the data on GTSP1 expression according to smoking and drinking habits. No differences were observed for the expression of GTSP1 in tumors between SD and NSND patients. An increase in the expression of GTSP1 was observed in the non-tumor margins of SD patients (p = 0.004).

**Table 2:** GTSP1 expression according to smoking and drinking habits. A: GSTP1 expression in non-tumor margins; B: GSTP1 expression in tumor.

A					
**GTSP1non-tumor margin**	SD low	SD high		total	*p*¥
NSND low	1 (6.3%)^§^	9 (56.3%)^¶^		10 (62.5%)	0.004
NSND high	0^¶^	6 (37.5%)^§^		6 (37.5%)	
B					
**GTSP1 tumor**	SD low	SD high		total	*p*¥
NSND low	0^§^	2 (6.7%)^¶^		2 (6.7%)	1
NSND high	1 (3.3%) ^¶^	27 (90.0%)^§^		28 (93.3%)	

¥McNemar’s test–each unit represents a pair of NSND and SD patients;

^§^concordant pairs (high-high or low-low)

^¶^discordant pairs (high-low or low-high)

No differences were observed for HPV positivity between NSND and SD patients. In addition, we found no association between HPV positivity and expression of GTSP1 among NSND patients or among SD patients [S1–S5 Tables].

**Fig 2:**
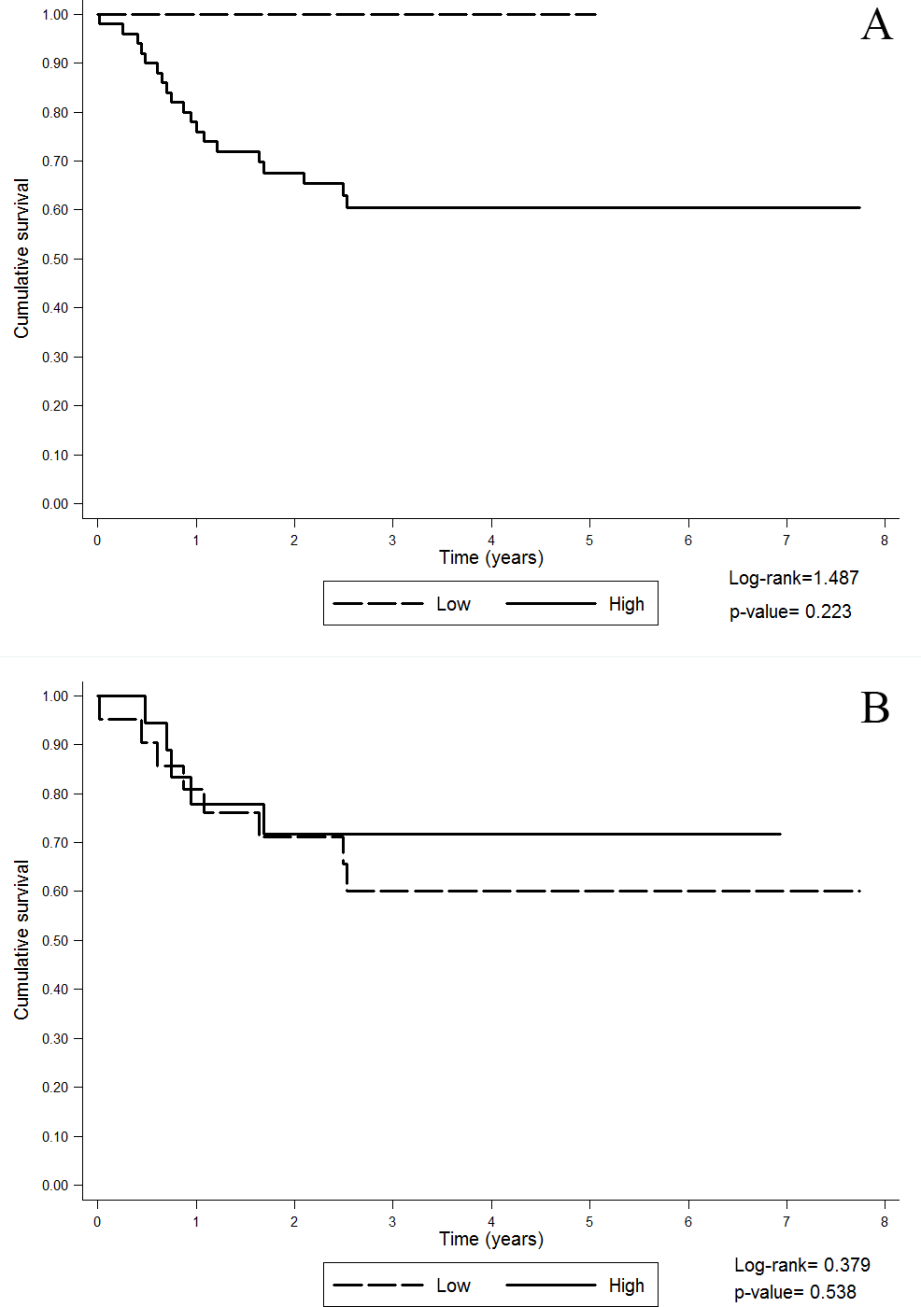
Overall survival of NSND patients according to GTSP1 expression. A: GTSP1 expression in non-tumor margins; B: GTSP1 expression in tumors.

There were no significant differences between the survival of NSND patients with high or low GTSP1 expression in either tumor or non-tumor margins (Fig 2A and 2B), even though the survival curves shown in Fig 2B seemed very different (Fig 2B).pression might have jeopardized the detection of a difference in survival according to GSTPI expressiogin

Original data are provided as supporting information (S6 and S7 Tables).

## Discussion

This is the first study to analyze the expression of GTSP1 in NSND patients with HNSCC. We detected GTSP1 expression in both tumors and non-tumor margins of patients with HNSCC irrespective of their drinking and smoking habits. However, GTSP1 expression was higher in the non-tumor margins of SD patients, which reinforces the detoxifying role of GTSP1 and indicates GTSP1 as a biomarker of exposure of non-neoplastic tissues to carcinogens.

The method of matched paired analysis enabled us to control for several possible confounders and analyze the data from patients with comparable clinical features who were only distinct in their exposure to carcinogens such as alcohol and tobacco. This information is not usually reported in the literature, as smoking and drinking habits are the most prevalent risk factors for HNSCC.

GTSP1 expression in the non-tumor margins was higher in the SD group compared to that in the NSND group. However, we cannot infer whether the exposure of the mucosal tissues of these patients to carcinogens was higher or lower than that of the mucosal tissues of healthy individuals. These data can be interpreted in two possible ways: First, there may have been an increase in GTSP1 expression in the mucosa of SD patients in response to the high degree of exposure to carcinogens. Second, alternatively, the reduction in the expression of GTSP1 in the mucosa of NSND patients could represent a risk factor for the development of cancer in this subset of patients. The relationship among GTSP1 expression, carcinogenesis and smoking and drinking habits is still unclear [8, 17, 26, 27], but we believe that our results reinforce the hypothesis that the status of GTSP1 should be analyzed in addition to the information regarding smoking and drinking habits in patients. Analysis of the genetic polymorphisms and enzymatic function of GTSP1 in these patients as well as in healthy subjects may clarify our findings. Polymorphisms in the GTSP1 gene have been associated with a reduction in the detoxifying capacity of the enzyme [8, 9, 28–31]. GSTs are involved in the detoxification of several drugs [32] and molecules related to oxidative stress; the generation of oxidative stress is an important antineoplastic mechanism of radiotherapy. An increase in the expression of GSTs might be partially responsible for resistance to chemotherapy in several tumors [11, 14, 24, 33, 34]. In contrast, genetic silencing of GTSP1 can increase the sensitivity of tumor cells to antineoplastic drugs, suggesting that low GTSP1 expression might be related to increased survival due to better response to treatment [16, 35]. Thus, GTSP1 might be not only a prognostic marker but also a predictor of response to treatment, and GTSP1 levels may thus guide treatment decisions. Studies on the prognostic value of GTSP1 in HNSCC have demonstrated worse prognosis in patients with increased expression of GTSP1 in tumors. However, these studies did not differentiate SD from NSND patients [9, 30, 36]. For this reason, we specifically analyzed the overall survival of NSND patients according to GTSP1 expression. Although the survival curves for patients with high and low tumoral expression of GTSP1 were visually distinct, the difference was not significant. This may have been because of the small number of patients with low expression of GTSP1 and because of the low rate of mortality among these patients.

The lack of association between HPV positivity and smoking and drinking habits might be explained by the fact that HPV-related cancers are not very common in Brazil [37]. Indeed, there is a geographical heterogeneity in HPV-related HNSCC worldwide [38]. Additionally, patients were recruited from the departments of head and neck surgery at the hospitals participating in this study. HPV usually affects the oropharynx, and tumors in these sites are frequently treated using non-surgical modalities with good response. In our study sample, most patients had tumors of the oral cavity, where HPV plays a minor and still unclear role.

### Limitations

The main limitation of our study was the small number of matched pairs, since HNSCC is less common in NSND individuals. Additionally, several NSND patients were elderly females with oral cavity tumors [18] who were difficult to match, because it is uncommon to find elderly women who smoke and drink. This might have been responsible for an increase in type 2 errors in our analyses. Nevertheless, these difficulties reinforced the effectiveness of the pairing method in controlling for confounders. A larger population size than that available from our consortium with more than 1600 patients is necessary to increase the power of our analyses. However, while challenging, the study on NSND patients is also a unique strength of this study. Since we did not study healthy subjects, we could not demonstrate whether GTSP1 is directly involved in the development of HNSCC. In this regard, this study primarily proposes the hypothesis that the detoxification of carcinogens is likely disrupted in NSND patients.

## Conclusion

Although studies investigating the polymorphisms in GTSP1 and other in vitro/in vivo studies are necessary to confirm our hypothesis, we have identified that the metabolic disturbance of carcinogen detoxification in NSND patients with HNSCC may be a possible risk factor for the development of cancer in patients without known risk factors. Additionally, if the rationale for the use of GTSP1 as a prognostic and predictive marker is further confirmed by other studies, the results should be interpreted in light of the information on the smoking and drinking habits of patients because these factors are directly related to GTSP1 expression, as demonstrated by our data.

## Supporting information

S1 Table Analysis of the association of HPV in matched patients NSND and SD. ^¥^McNemar, NSND: non-smokers and non-drinkers; SD: smokers and drinkers; HPV: human papillomavirus.
(PDF)

S2 Table Analysis of the association of HPV according to the expression of GSTPI in the margin of NSND patients. ^¥^Fisher's exact test NSND: non-smokers and non-drinkers; HPV: human papillomavirus.
(PDF)

S3 Table Analysis of the association of HPV according to the expression of GSTPI in the tumor of NSND patients. ^¥^Fisher's exact test NSND: non-smokers and non-drinkers; HPV: human papillomavirus.
(PDF)

S4 Table Analysis of the association of HPV according to the expression of GSTPI in the margin of SD patients. ^¥^Fisher's exact test SD: smokers and drinkers; HPV: human papillomavirus.
(PDF)

S5 Table Analysis of the association of HPV according to the expression of GSTPI in the tumor of SD patients. ^¥^Fisher's exact test SD: smokers and drinkers; HPV: human papillomavirus.
(PDF)

S6 Table Data of paired subjects. (PDF)

S7 Table Data for survival analysis—NSND subjects. (PDF)
