# Supplementary material for: GTSP1 expression in non-smoker and non-drinker patients with squamous cell carcinoma of the head and neck
Source: PLoS One. 2017 Aug 17;12(8):e0182600. doi: 10.1371/journal.pone.0182600 (PMC5560606; doi:10.1371/journal.pone.0182600)
Supplement: S1 Table — ¥McNemar, NSND: non-smokers and non-drinkers; SD: smokers and drinkers; HPV: human papillomavirus. (PDF) [file pone.0182600.s001.pdf]

S1 Table. Analysis of the association of HPV in matched patients NSND and SD

		SD		p¥
NSND	HPV	HPV negative	HPV positive	0.180
	negative	24 (92.3%)	2 (7.7%)	
	positive	7(100%)	0	

¥McNemar, NSND: non-smokers and non-drinkers; SD: smokers and drinkers; HPV: human papillomavirus
