# Supplementary material for: GTSP1 expression in non-smoker and non-drinker patients with squamous cell carcinoma of the head and neck
Source: PLoS One. 2017 Aug 17;12(8):e0182600. doi: 10.1371/journal.pone.0182600 (PMC5560606; doi:10.1371/journal.pone.0182600)
Supplement: S2 Table — ¥Fisher's exact test NSND: non-smokers and non-drinkers; HPV: human papillomavirus. (PDF) [file pone.0182600.s002.pdf]

**S2 Table. Analysis of the association of HPV according to the expression of GSTPI in the margin of NSND patients**

| | | GSTPI margin | | $p^{¥}$ |
|---|---|---|---|---|
| NSND | **HPV** | **low** | **high** | |
| | **negative** | 10 (62.5%) | 6 (37.5%) | 1 |
| | **positive** | 3 (60%) | 2 (40%) | |

¥*Fisher's exact test NSND: non-smokers and non-drinkers; HPV: human papillomavirus*
