# Supplementary material for: GTSP1 expression in non-smoker and non-drinker patients with squamous cell carcinoma of the head and neck
Source: PLoS One. 2017 Aug 17;12(8):e0182600. doi: 10.1371/journal.pone.0182600 (PMC5560606; doi:10.1371/journal.pone.0182600)
Supplement: S3 Table — ¥Fisher's exact test NSND: non-smokers and non-drinkers; HPV: human papillomavirus. (PDF) [file pone.0182600.s003.pdf]

S3 Table. Analysis of the association of HPV according to the expression of GSTPI in the tumor of NSND patients

		GSTPI tumor		p[¥]
NSND	HPV	low	high	0.355
	negative	1 (4%)	24 (96.0%)	
	positive	1 (16.7%)	5 (83.3%)	

[¥]*Fisher's exact test NSND: non-smokers and non-drinkers; HPV: human papillomavirus*
