# Supplementary material for: GTSP1 expression in non-smoker and non-drinker patients with squamous cell carcinoma of the head and neck
Source: PLoS One. 2017 Aug 17;12(8):e0182600. doi: 10.1371/journal.pone.0182600 (PMC5560606; doi:10.1371/journal.pone.0182600)
Supplement: S4 Table — ¥Fisher's exact test SD: smokers and drinkers; HPV: human papillomavirus. (PDF) [file pone.0182600.s004.pdf]

S4 Table. Analysis of the association of HPV according to the expression of GSTPI in the margin of SD patients

		GSTPI margin		$p^{¥}$
SD	HPV	low	high	1
	negative	4 (20%)	16 (80%)	
	positive	0	1 (100%)	

¥Fisher's exact test SD: smokers and drinkers; HPV: human papillomavirus
