# Supplementary material for: GTSP1 expression in non-smoker and non-drinker patients with squamous cell carcinoma of the head and neck
Source: PLoS One. 2017 Aug 17;12(8):e0182600. doi: 10.1371/journal.pone.0182600 (PMC5560606; doi:10.1371/journal.pone.0182600)
Supplement: S5 Table — ¥Fisher's exact test SD: smokers and drinkers; HPV: human papillomavirus. (PDF) [file pone.0182600.s005.pdf]

S5 Table. Analysis of the association of HPV according to the expression of GSTPI in the tumor of SD patients

		GSTPI tumor		$p^{¥}$
SD	**HPV**	**low**	**high**	x
	**negative**	0	20 (100%)	
	**positive**	1	0	

¥Fisher's exact test SD: smokers and drinkers; HPV: human papillomavirus
