# Supplementary material for: GTSP1 expression in non-smoker and non-drinker patients with squamous cell carcinoma of the head and neck
Source: PLoS One. 2017 Aug 17;12(8):e0182600. doi: 10.1371/journal.pone.0182600 (PMC5560606; doi:10.1371/journal.pone.0182600)
Supplement: S6 Table — (PDF) [file pone.0182600.s006.pdf]

id1	id2	hab1	hab2	sex1	sex2	age1	age2	Ethnicity1
6	47	NSND	SD	2	2	71	70	White
7	46	NSND	SD	1	1	73	75	Mulatto
8	99	NSND	SD	1	1	71	75	White
9	1	NSND	SD	1	1	70	70	White
14	18	NSND	SD	2	2	56	56	White
15	48	NSND	SD	1	1	61	59	Black
17	25	NSND	SD	2	2	76	80	White
19	73	NSND	SD	2	2	71	70	White
21	87	NSND	SD	2	2	39	42	White
26	4	NSND	SD	1	1	40	39	White
27	38	NSND	SD	1	2	54	56	White
28	53	NSND	SD	1	1	35	39	White
30	86	NSND	SD	1	1	57	61	White
32	89	NSND	SD	2	2	50	53	White
34	33	NSND	SD	2	2	73	70	White
36	79	NSND	SD	1	1	42	39	White
37	59	NSND	SD	1	1	63	63	White
40	83	NSND	SD	1	1	58	57	Black
41	76	NSND	SD	1	1	73	70	White
44	77	NSND	SD	1	1	51	50	White
50	77	NSND	SD	1	1	51	50	White
51	81	NSND	SD	2	2	69	66	White
52	3	NSND	SD	1	1	55	54	Black
54	33	NSND	SD	2	2	66	70	White
55	22	NSND	SD	2	2	63	63	White
56	107	NSND	SD	1	1	58	61	White
58	76	NSND	SD	1	1	66	70	White
62	2	NSND	SD	1	1	65	68	Asian
63	61	NSND	SD	1	1	81	79	White
65	64	NSND	SD	2	2	36	41	99
68	61	NSND	SD	1	1	77	79	White
72	16	NSND	SD	1	1	69	71	White
75	29	NSND	SD	1	1	65	68	White
78	76	NSND	SD	1	1	70	70	White
88	23	NSND	SD	2	2	52	51	White
90	61	NSND	SD	1	1	79	79	White
91	61	NSND	SD	1	1	80	79	Asian
92	29	NSND	SD	1	1	66	68	White
93	61	NSND	SD	1	1	83	79	White
94	12	NSND	SD	2	2	56	53	White
95	1	NSND	SD	1	1	74	70	White
96	67	NSND	SD	2	2	52	50	Asian
97	46	NSND	SD	1	1	74	75	White
103	84	NSND	SD	2	2	52	51	White
104	23	NSND	SD	2	2	53	51	Mulatto
106	1	NSND	SD	1	1	72	70	White
109	31	NSND	SD	2	2	74	69	White

Ethnicity2	t1	t2	n1	n2	site1	site2	tumor varia	tumor varia
White	4	4	1	1	5	5	1	1
White	2	2	1	1	1	1	1	1
White	2	2	0	0	1	1	3	2
White	3	4	0	0	1	1	1	2
White	4	4	1	1	2	2	1	1
White	1	1	0	0	1	1	1	1
White	4	4	0	0	4	4	1	1
White	2	2	0	0	1	1	1	1
Black	4	4	0	0	4	4	1	1
Mulatto	3	4	1	1	1	1	1	1
Mulatto	4	4	1	1	1	1	1	1
White	1	1	0	0	1	1	1	1
Mulatto	4	3	0	0	1	1	1	1
White	2	2	0	0	3	3	1	1
White	1	1	0	0	4	4	1	1
Mulatto	1	1	0	0	1	1	1	1
White	2	3	0	0	4	4		1
White	2	1	0	0	1	1	1	1
Black	2	2	0	0	1	1	1	1
Mulatto	2	1	1	1	1	1	1	1
Mulatto	2	1	1	1	1	1	1	1
White	1	1	1	1	1	1	1	1
Mulatto	4	3	0	0	4	4	1	1
White	1	1	0	0	4	4	1	1
Mulatto	3	3	1	1	1	1	1	1
White	1	2	0	0	4	4	1	
Black	2	2	0	0	1	1	1	1
Mulatto	4	4	0	0	1	1	1	1
White	2	1	0	0	1	1	1	1
White	1	1	0	0	1	1	1	1
White	2	1	0	0	1	1	1	1
Mulatto	1	1	0	0	1	1	1	1
White	3	4	1	1	1	1	1	1
Black	2	2	0	0	1	1	1	1
White	4	4	1	1	4	4	1	1
White	2	1	0	0	1	1	1	1
White	1	1	0	0	1	1	1	1
White	4	4	1	1	1	1	1	1
White	2	1	0	0	1	1	1	1
Mulatto	3	4	1	1	2	2	1	1
White	4	4	0	0	1	1	1	2
White	4	4	1	1	4	4	1	1
White	2	2	1	1	1	1	1	1
Black	2	2	1	1	1	1		1
White	4	4	1	1	4	4		1
White	2	4	0	0	1	1		2
White	3	4	1	1	1	1		1

grade1	grade2	hpv1	hpv2	pair1	pair2	GSTPi_marginNSND
1	2	0	0	1	1	99
1	2	0	0	2	2	0
1	1			3	3	99
1	2	0	0	4	4	0
1	1	0	0	5	5	1
2	2	1	0	6	6	0
1	1		0	7	7	99
2	2	1	0	8	8	99
1	2		0	9	9	99
1	2	1	0	10	10	1
1	1	1	0	11	11	99
	1		0	12	12	99
2	2	0	1	13	13	99
2	2	0	0	14	14	0
1	1	0	0	15	15	99
2	2	0	0	16	16	1
1	1	0	0	17	17	99
1	1	0	0	18	18	99
2	1		0	19	19	99
2	2	0	0	20	20	0
1	2	0	0	21	21	0
2	1	0	1	22	22	0
2	2	1		23	23	99
1	1	0	0	24	24	99
2	2	0	0	25	25	99
	2	1		26	26	99
1	1	0	0	27	27	0
2	2	1	0	28	28	0
1	1	1	0	29	29	0
1	2	0	0	30	30	99
1	1	1	0	31	31	1
1	1	0	0	32	32	0
1	2	0		33	33	1
2	1	0	0	34	34	1
1	1	0	0	35	35	99
1	1	0	0	36	36	0
1	1	0	0	37	37	1
1	2	1		38	38	99
2	1	0	0	39	39	1
1	2	0	0	40	40	99
2	2	0	0	41	41	0
3	3	1		42	42	99
2	2	0	0	43	43	99
2	2		0	44	44	99
1	1		0	45	45	99
1	2		0	46	46	99
1	1		0	47	47	99

GSTPi_marginSD	GSTPi_tumorNSND	GSTPi_tumorSD
99	1	1
0	1	1
99	99	99
1	1	1
1	1	1
99	0	1
1	1	1
1	99	0
99	1	1
99	1	0
0	1	1
0	99	99
1	0	1
99	1	99
1	1	1
1	1	1
1	1	1
1	1	1
1	99	1
1	1	1
1	1	1
99	1	1
99	99	99
1	1	1
0	1	1
99	99	99
1	1	1
1	1	1
1	1	1
99	1	99
1	1	1
1	1	1
99	1	99
1	1	1
0	99	1
1	1	1
1	1	1
99	99	99
1	1	1
1	1	1
1	1	1
99	99	99
0	99	1
0	99	1
0	99	1
1	99	1
1	99	1
