# Supplementary material for: GTSP1 expression in non-smoker and non-drinker patients with squamous cell carcinoma of the head and neck
Source: PLoS One. 2017 Aug 17;12(8):e0182600. doi: 10.1371/journal.pone.0182600 (PMC5560606; doi:10.1371/journal.pone.0182600)
Supplement: S7 Table — (PDF) [file pone.0182600.s007.pdf]

ID	hab1	age1	t1	n1	site1	variant1	grade1	pair1	time_days	GSTPi_marginNSND	GSTPi_tumorNSND
6	NSND	71	4	1	5	1	1	1	239	99	1
7	NSND	73	2	1	1	1	1	2	1783	0	1
8	NSND	71	2	0	1	3	1	3	317	99	99
9	NSND	70	3	0	1	1	1	4	597	0	1
14	NSND	56	4	1	2	1	1	5	748	1	1
15	NSND	61	1	0	1	1	2	6	1522	0	0
17	NSND	76	4	0	4	1	1	7	1372	99	1
19	NSND	71	2	0	1	1	2	8	1	99	99
21	NSND	39	4	0	4	1	1	9	794	99	1
26	NSND	40	3	1	1	1	1	10	257	1	1
27	NSND	54	4	1	1	1	1	11	149	99	1
28	NSND	35	1	0	1	1		12	2275	99	99
30	NSND	57	4	0	1	1	2	13	1847	99	0
32	NSND	50	2	0	3	1	2	14	1714	0	1
34	NSND	73	1	0	4	1	1	15	508	99	1
36	NSND	42	1	0	1	1	2	16	1524	1	1
37	NSND	63	2	0	4		1	17	1474	99	1
40	NSND	58	2	0	1	1	1	18	764	99	1
41	NSND	73	2	0	1	1	2	19	1092	99	99
44	NSND	51	2	1	1	1	2	20	2826	0	1
50	NSND	51	2	1	1	1	1	21	318	0	1
51	NSND	69	1	1	1	1	2	22	221	0	1
52	NSND	55	4	0	4	1	2	23	2389	99	99
54	NSND	66	1	0	4	1	1	24	2186	99	1
55	NSND	63	3	1	1	1	2	25	368	99	1
56	NSND	58	1	0	4	1		26	2248	99	99
58	NSND	66	2	0	1	1	1	27	2174	0	1
62	NSND	65	4	0	1	1	2	28	1977	0	1
63	NSND	81	2	0	1	1	1	29	925	0	1
65	NSND	36	1	0	1	1	1	30	1755	99	1
68	NSND	77	2	0	1	1	1	31	1406	1	1
72	NSND	69	1	0	1	1	1	32	1315	0	1
75	NSND	65	3	1	1	1	1	33	176	1	1
78	NSND	70	2	0	1	1	2	34	617	1	1
88	NSND	52	4	1	4	1	1	35	361	99	99
90	NSND	79	2	0	1	1	1	36	853	0	1
91	NSND	80	1	0	1	1	1	37	540	1	1
92	NSND	66	4	1	1	1	1	38	499	99	99
93	NSND	83	2	0	1	1	2	39	346	1	1
94	NSND	56	3	1	2	1	1	40	92	99	1
95	NSND	74	4	0	1	1	2	41	439	0	1
96	NSND	52	4	1	4	1	3	42	439	99	99
97	NSND	74	2	1	1	1	2	43	95	99	99
103	NSND	52	2	1	1		2	44	268	99	99
104	NSND	53	4	1	4		1	45	1313	99	99
106	NSND	72	2	0	1		1	46	371	99	99
109	NSND	74	3	1	1		1	47	342	99	99

5	NSND	70	2	1	3	1	2	1970	1	1
10	NSND	81	4	0	1	1	1	1270	1	0
11	NSND	79	3	0	1	1	2	1249	1	1
13	NSND	85	4	0	1	1	2	1638	1	1
20	NSND	79	4	1	1	1	1	163	0	1
24	NSND	41	1	1	1	1	3	1918	99	99
35	NSND	75	4	1	1	1	1	911	0	1
39	NSND	80	4	0	1	1	2	443	99	1
42	NSND	25	2	1	1	1	2	2465	1	1
43	NSND	21	0	1	3	1	1	172	99	99
45	NSND	38	1	0	3	1	1	2784	0	1
49	NSND	93	4	0	1	1	1	5	0	1
57	NSND	75	3	0	2	3		2233	99	1
60	NSND	48	1	0	3	1	1	1980	1	1
66	NSND	49	1	0	3	1	2	395	0	1
69	NSND	55	3	0	1	1	1	1487	0	1
70	NSND	86	2	0	1	1	2	12	99	99
71	NSND	62	2	1	1	1	2	1610	0	1
74	NSND	29	2	1	2	1	3	1426	1	1
80	NSND	72		1	3	1	2	307	99	99
82	NSND	81	2	1	1	1	2	272	1	1
85	NSND	28	2	1	3	1	2	1113	0	1
98	NSND	68	2	0	3	4	1	1	0	1
100	NSND	56	1	1	2		3	2532	1	1
101	NSND	92	2	0	3		1		0	1
102	NSND	27	1	0	1		2		1	1
105	NSND	63	4	1	1		3	138	99	99
108	NSND	64	4	0	2		1	1847	99	99
110	NSND	58			4	2		1104	1	1
